# Human bone marrow stem/stromal cell osteogenesis is regulated via mechanically activated osteocyte‐derived extracellular vesicles

**DOI:** 10.1002/sctm.19-0405

**Published:** 2020-07-16

**Authors:** Kian F. Eichholz, Ian Woods, Mathieu Riffault, Gillian P. Johnson, Michele Corrigan, Michelle C. Lowry, Nian Shen, Marie‐Noelle Labour, Kieran Wynne, Lorraine O'Driscoll, David A. Hoey

**Affiliations:** ^1^ Department of Mechanical, Aeronautical and Biomedical Engineering Materials and Surface Science Institute, University of Limerick Limerick Ireland; ^2^ Trinity Centre for Biomedical Engineering Trinity Biomedical Sciences Institute, Trinity College Dublin Dublin 2 Ireland; ^3^ Department of Mechanical and Manufacturing Engineering School of Engineering, Trinity College Dublin Dublin 2 Ireland; ^4^ School of Pharmacy and Pharmaceutical Sciences and Trinity Biomedical Sciences Institute Trinity College Dublin Dublin Ireland; ^5^ UCD Conway Institute of Biomolecular and Biomedical Research University College Dublin Dublin 4 Ireland; ^6^ Mass Spectrometry Resource University College Dublin Dublin 4 Ireland; ^7^ Advanced Materials and Bioengineering Research Centre Trinity College Dublin & RCSI Dublin Ireland

**Keywords:** bone, extracellular vesicle, marrow stem cell, mechanobiology, proteomics

## Abstract

Bone formation or regeneration requires the recruitment, proliferation, and osteogenic differentiation of stem/stromal progenitor cells. A potent stimulus driving this process is mechanical loading. Osteocytes are mechanosensitive cells that play fundamental roles in coordinating loading‐induced bone formation via the secretion of paracrine factors. However, the exact mechanisms by which osteocytes relay mechanical signals to these progenitor cells are poorly understood. Therefore, this study aimed to demonstrate the potency of the mechanically stimulated osteocyte secretome in driving human bone marrow stem/stromal cell (hMSC) recruitment and differentiation, and characterize the secretome to identify potential factors regulating stem cell behavior and bone mechanobiology. We demonstrate that osteocytes subjected to fluid shear secrete a distinct collection of factors that significantly enhance hMSC recruitment and osteogenesis and demonstrate the key role of extracellular vesicles (EVs) in driving these effects. This demonstrates the pro‐osteogenic potential of osteocyte‐derived mechanically activated extracellular vesicles, which have great potential as a cell‐free therapy to enhance bone regeneration and repair in diseases such as osteoporosis.

## Graphical abstract

Bone formation/regeneration requires the recruitment and osteogenic differentiation of stem/stromal progenitor cells. A potent stimulus driving this process is mechanical loading. In his study, we demonstrate that osteocytes coordinate stem cell behavior in response to loading via modulation of their secretome that is delivered via extracellular vesicles (EVs). This therefore highlights mechanically activated EVs as potential therapies for bone regeneration.

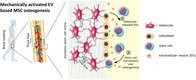

**Significance statement**

Bone regeneration requires the osteogenesis of stem/stromal progenitor cells. A potent stimulus driving this process is physical loading. Osteocytes are mechanosensitive cells which play fundamental roles in coordinating bone mechanoadaptation. However, the exact mechanisms are poorly understood. This study demonstrates that osteocytes subjected to fluid shear secrete a distinct collection of factors that enhance stem cell recruitment and osteogenesis. Moreover, this study identified that these factors are delivered via extracellular vesicles (EVs), demonstrating a novel mechanism of osteocyte‐stem cell communication. Therefore, osteocyte‐derived mechanically activated EVs hold great potential as a novel cell‐free therapy to enhance bone regeneration.

## 1 INTRODUCTION

Osteocytes are the most abundant cell type in bone and are known as the primary sensing and metabolism‐controlling cells within the tissue. Osteocytes are key to directing the processes of bone formation and resorption via the secretion of various signaling factors which act upon bone forming osteoblasts and resorbing osteoclasts and their progenitors, skeletal, and hematopoietic stem cells.^1^ The implications of this can be seen in the highly debilitating and life‐threatening disease that is osteoporosis, which has been linked to osteocyte apoptosis^2^ and reduced osteocyte numbers in affected patients.^3^ This results in a significant drop in quality of life, increased risk of additional complications due to immobilization, and significantly increased mortality rates due to fracture and secondary causes.^4^ Not only do osteocytes have key functions in bone, but they have also been shown to be involved in a large range of other major functions throughout the body,^5^ including heart, muscle and liver function, and suppressing breast cancer growth and metastasis in bone.^6^ This highlights the critical role of the osteocyte in human health, and the importance of better understanding osteocyte signaling factors for the development of therapeutics to treat orthopedic and systemic diseases.

A prime example of osteocyte sensing and coordination of bone physiology is in mechanoadaptation, with mechanical loading leading to enhanced bone formation and unloading leading to bone loss.^7^ In response to macroscale deformation of bone, resident osteocytes sense the micromechanical environment consisting of oscillatory fluid flow‐induced shear stress and relay this biophysical signal to effector cells.^8^ Mechanically stimulated osteocytes can enhance the bone forming capacity of osteoblasts via direct cell‐cell contact,^9^ in addition to secreted factors as demonstrated by conditioned media experiments.^10^, ^11^ Furthermore, this same mechanically activated osteocyte conditioned media was also shown to inhibit osteoclast formation.^12^, ^13^ Due to the nonproliferative state and short life span of mature bone cells, continuous bone formation requires the replenishment of the exhausted osteoblast from a stem cell or progenitor population.^14^ Interestingly, the osteocyte has also been shown to coordinate bone marrow stem/stromal cell (MSC) behavior, with conditioned media from mechanically stimulated osteocytes enhancing stem cell proliferation, recruitment, and osteogenic differentiation, demonstrating the far reaching influence of this cell type, particularly in response to a mechanical stimulus.^10^

The means by which osteocytes coordinate this mechanoadaptation of bone is of great interest, with several key factors identified as playing a role in this regard and, therefore, targeted as therapeutics. There has been a plethora of studies investigating various osteocyte‐derived factors released in response to fluid shear, including nitric oxide (NO), prostaglandin E2, ATP, RANKL, osteoprotegerin (OPG), and macrophage colony‐stimulating factor (M‐CSF).^1^ One factor that has gained much interest is sclerostin (SOST) which is released by osteocytes and inhibits Wnt‐mediated bone formation. SOST expression is inhibited following mechanical loading and inhibition of this protein via anti‐sclerostin therapy has been shown in clinical trials to increase bone mineral density and reduce fracture risk.^15^ To gain a greater understanding of the factors expressed by physically stimulated osteocytes, others have taken a more global approach, using microarrays to study global gene expression in osteocytes subjected to cyclic compressive forces^16^ and osteocytes isolated from murine trabecular bone following vertebrae loading.^17^ Furthermore, a proteomic analysis has been combined with a transcriptomic analysis of osteocytes subjected to fluid shear to investigate protein as well as gene expression information and reveal novel interactions between them.^18^ These studies revealed the altered proteome of the osteocyte, due to fluid flow stimulation, and identified a range of proteins which may be involved in mechanotransduction, including nucleoside diphosphate kinase and calcyclin, which are of interest due to their roles in ATP and calcium‐binding, respectively. However, to date, the full secretome protein signature of the osteocyte and how this is altered in response to mechanical stimulation is unknown.

A route of cell‐cell communication which has garnered much attention of late is via extracellular vesicles (EVs). EVs are spherical proteolipids bilayer surrounded vesicles secreted from cells and are involved in cell‐cell communication. EVs can transfer cargo including lipids, proteins, and nucleic acid from one cell to another, thereby influencing the recipient cell function.^19^ Interestingly, it has recently been shown that bone cells release EVs and use these vesicles as a mechanism to mediate osteoblast and stem cell osteogenesis.^20^, ^21^, ^22^, ^23^, ^24^ Moreover, osteocyte‐derived EVs contain miRNAs known to mediate osteoblast function, highlighting a potential nonprotein based role in bone cell communication.^25^, ^26^ Bone derived EVs may also be exploited as a potential therapy for various diseases, as well as having potential for treatment of critical size bone defects.^27^ In addition, the capability to load EVs with factors to guide cell behavior both in vitro and in vivo has been shown,^28^ supporting their potential as a powerful drug delivery method. Interestingly, the release of EVs—and thus their content—may also be altered by mechanical loading. In fact, EV release into plasma increases following exercise, with a differential protein cargo in EVs from subjects after exercise compared to those at rest.^29^ In summary, the importance of the role EVs play in osteogenesis can be seen, and due to the dynamic nature of this tissue, indicate potential further mechanisms involving EVs in bone mechanoadaptation.

While changes in several factors in and released by osteocytes have been shown via proteomics analysis, the specific composition and factors implicated in mechanically mediated osteocyte paracrine signaling are yet to be elucidated. Thus, the aim of this study was to further investigate the means by which osteocytes mediate bone mechanoadaptation, with this being achieved by constructing, for the first time, an extensive map of the osteocyte secretome protein signature. This map comprises the relative expression of hundreds of proteins in the osteocyte proteome under static and dynamic conditions in addition to providing information on how they interact with one another. We first validated the ability of the osteocyte secretome to induce a chemotactic and osteogenic response in human bone marrow stem/stromal cells (hMSCs) using a parallel plate flow chamber approach to mechanically stimulate osteocytes. We then conducted a proteomic analysis on the osteocyte secretome via mass spectrometry, to identify proteins released by cells under both static and dynamic culture conditions. Enrichment of gene ontology terms was investigated to elucidate the primary cellular components and processes with which the osteocyte secretome is involved, with further analysis comparing the altered protein release and most differentially expressed proteins released by mechanically stimulated cells. This led to the discovery of MA‐EVs. Specifically, EVs were subsequently separated from the secretome of mechanical‐activated osteocyte; characterised; and found to elicit similar trends in MSC recruitment and osteogenesis to that seen with conditioned media (ie, whole secretome). We further demonstrated how MA‐EVs drove enhanced later term osteogenesis as assessed by alkaline phosphatase (ALP) activity. This demonstrated a key role for osteocyte EVs in mediating hMSC behavior, identifying a potential novel mechanism by which osteocytes coordinate loading‐induced bone formation.

## 2 MATERIALS AND METHODS

### 2.1 Cell culture

MLO‐Y4 osteocyte like cells (Kerafast)^30^ were cultured, as previously described,^31^ in α‐MEM growth medium with 2.5% fetal bovine serum (FBS, lot #11307, Biosera), 2.5% calf serum (CS, lot #10406, Biosera), 1% penicillin/streptomycin (PS), and 1% L‐glutamine during static culture and fluid shear stimulation. For whole secretome/conditioned medium (CM) studies, cells were cultured in α‐MEM with 1% PS and 1% L‐glutamine. hMSCs were isolated from bone marrow (Lonza), characterized by tri‐lineage differentiation (data not shown), and maintained in Dulbecco's modified eagle medium with 10% FBS and 1% PS unless otherwise stated. All cells were cultured at 37°C and 5% CO_2_.

### 2.2 Mechanical stimulation and conditioned medium collection

Forty‐eight hours prior to fluid shear application, 75 × 38 mm glass slides were coated with 0.15 mg/mL type I collagen (Sigma C3867) for 1 hour and washed with phosphate buffered saline (PBS), after which osteocytes were seeded at a density of 1.16 × 10^4^ cells/cm^2^. Glass slides were transferred to custom‐made parallel plate flow chambers (PPFCs) as previously described.^32^ Each glass slide was assembled within an individual PPFC under sterile conditions and incubated at 37°C and 5% CO_2_. Cells in PPFCs were either subjected to a fluid shear stress of 1 Pa at a frequency of 1 Hz, or maintained in the PPFC under static conditions, with each condition completed in quadruplicate. After 2 hours of treatment, slides were transferred to culture dishes, washed with PBS, and 2.5 mL of serum‐free medium was applied. A control group consisting of collagen‐coated glass slides with no cells was also incubated with 2.5 mL of serum‐free medium. All culture dishes were incubated for 24 hours and medium was collected from cells which had undergone fluid shear (CM‐F), statically cultured cells (CM‐S) and from cell‐free slides with collagen coating (Medium). Samples were centrifuged at 3000*g* for 10 minutes at 4°C to remove debris, after which the supernatant was collected and stored at −80°C prior to use (Figure 2A).

### 2.3 Effect of osteocyte conditioned media on bone marrow stem/stromal cell recruitment

Chemotaxis of hMSCs was assessed using Boyden chambers with a pore size of 8 μm (Merck Millipore, PIEP12R48). Cells were seeded on the upper membrane in serum‐free α‐MEM medium at a density of 30 000 cells/cm^2^ and allowed to adhere 4 hours before being transferred to the wells containing chemotactant (Medium [serum‐free], CM‐S, CM‐F, Medium + 10% FBS). Cells were then cultured for a further 18 hours, fixed with 10% formalin solution, and stained with hematoxylin. Light microscopy was used to determine the number of migrated cells, which was then normalized to Medium for each group.

### 2.4 Effect of osteocyte conditioned media on bone marrow stem/stromal cell osteogenesis

hMSC cells were seeded in 6‐well plates at a density of 6500 cells/cm^2^ and cultured for 24 hours. Osteocyte CM (CM‐S, CM‐F) was then applied and hMSCs were cultured for a further 24 hours after which time cells were lysed with tri‐reagent (Sigma Aldrich) and mRNA isolated as per the manufacturer's protocol. RNA concentration was measured using a Nanodrop spectrophotometer and sample purity was checked via 260/280 and 260/230 absorbance ratios. Two hundred nanograms RNA was reverse transcribed to cDNA using a High‐Capacity cDNA Reverse Transcription Kit (Applied Biosystems). Commercially available primers (Sigma Aldrich, KSPQ12012) were used to determine levels of cyclooxygenase 2 (COX2), osteocalcin (OCN), osteopontin (OPN), runt‐related transcription factor 2 (RUNX2), and osterix (OSX) (Table S1). Glyceraldehyde‐3‐Phosphate Dehydrogenase (GAPDH) and 18S were used as reference genes for this study. Quantitative polymerase chain reaction (qPCR) analysis was performed using a reaction volume of 20 μL containing 10 μL SYBR green PCR MasterMix (Invitrogen, Ltd, Paisley, UK), 0.8 μL of each forward and reverse primer, and 8.4 μL DNase‐free water. Plates were run on an ABI 7500 Fast real‐time PCR system (Life Technologies, Carlsbad, California).

### 2.5 Sample preparation for mass spectrometry (MS) analysis

Protein precipitation was carried out with 1 mL of each sample (Medium, CM‐S, CM‐F) using trichloroacetic acid, and, following centrifugation at 18 500*g*, the pellet resuspended in 6 M urea in 50 mM ammonium bicarbonate. Samples were reduced with 5 mM dithiothreitol for 30 minutes at 60°C and alkylated with 10 mM iodoacetamide for 30 minutes at room temperature in the dark, after which ammonium bicarbonate was added to bring the concentration of urea to 1.8 M. The reduced and alkylated proteins were then digested overnight with trypsin at a ratio of 1:50 wt/wt trypsin to protein at 37°C and 350 rpm on a Thermomixer. Digestion was then stopped with 8.8 M hydrochloric acid. Peptides were bound and desalted using C18 ZipTips (Merck Millipore) and washed with 0.1% trifluoroacetic acid (TFA) before being resuspended in 10 μL elution solution (50% acetonitrile in 0.1% TFA). Samples were concentrated using a SpeedVac vacuum concentrator until roughly 4 μL remained, before being resuspended in 20 μL 0.5% acetic acid (Figure 2B).

#### 2.5.1 *Liquid chromatography‐mass spectrometry and tandem mass spectrometry (LC MS/MS) analysis*

Biological samples (n = 3) were run with two technical replicates on a Thermo Scientific Q Exactive mass spectrometer connected to a Dionex Ultimate 3000 (RSLCnano) chromatography system. Each sample was loaded onto a fused silica emitter (75 μm ID, pulled using a laser puller [Sutter Instruments P2000]), packed with UChrom C18 (1.8 μm) reverse phase media (nanoLCMS Solutions LCC) and was separated by an increasing acetonitrile gradient over 47/60 minutes at a flow rate of 250 nL/min. The MS was operated in positive ion mode with a capillary temperature of 320°C, and with a potential of 2300 V applied to the frit. All data were acquired with the MS operating in automatic data‐dependent switching mode. A high resolution (70 000) MS scan (300‐1600 m/z) was performed using the Q Exactive to select the eight most intense ions prior to MS/MS analysis using high‐energy collision dissociation.

### 2.6 MS data analysis

Raw data from MS analysis was processed using MaxQuant software^33^, ^34^ version 1.5.5.1 and spectra searched using the built in Andromeda search engine^35^ with the Uniprot FASTA validated *Mus musculus* database being used as the forward database and the reverse for the decoy search being generated within the software. A minimum six amino acid length criteria was applied and the false discovery rate (FDR) for MS data analysis was set to 1% at the peptide and protein level. Cysteine carbamidomethylation was included as a fixed modification and oxidation of methionine and protein N‐terminal acetylation were set as variable modifications for the peptide search. The “match between runs” algorithm was used to transfer peptide identifications between MS runs where possible to increase total number of protein hits. At least one unique or razor peptide was required per protein group for identification. Label‐free quantification (LFQ) was carried out using the MaxLFQ algorithm^36^ within the software, with Fast LFQ being disabled. Other settings were kept as default in the software.

### 2.7 EV isolation from conditioned media

Medium from statically and dynamically cultured osteocytes was collected and centrifuged at 3000*g* for 10 minutes to remove debris. Medium was then filtered through a 0.45 μm pore filter. Medium was subsequently ultracentrifuged at 110 000*g* for 75 minutes at 4°C, using an SW32.Ti swing bucket rotor. Collected EV pellets were washed in PBS and the ultracentrifugation process was repeated.

### 2.8 Characterization of EVs

#### 2.8.1 *Transmission electron microscope imaging*

EV imaging was conducted via a JEOL JEM1400 transmission electron microscope (TEM) coupled with an AMT XR80 digital acquisition system. Samples were physiosorbed to 200 mesh carbon‐coated copper formvar grids and negatively stained with 1% uranyl acetate.

#### 2.8.2 *Immunoblotting*

For immunoblotting, cell pellets and EVs were lysed using cell extraction buffer (Invitrogen, Carlsbad, California) supplemented with protease inhibitor cocktail (Roche, Basel, Switzerland). Protein quantification was performed using Bio‐Rad protein assay (Bio‐Rad, Hercules, California). Cellular and EV protein (8 μg) were resolved on 10% SDS gels and transferred to polyvinylidene difluoride (PVDF) membranes (BioRad). Blots were incubated at 4°C overnight with monoclonal primary antibodies to GRP‐94 (clone D6X2Q, Rabbit mAb #20292s from Cell Signaling, 1:2000 dilution), TSG101 (clone 4A10, mouse mAb #ab83 from Abcam, 1:1000 dilution), and PDC6I/ALIX (clone 3A9, mouse mAb #ab117600 from Abcam, 1:1000 dilution). Secondary antibodies were incubated for 1 hour at room temperature and developed using Immobilon Western Chemiluminescent horseradish peroxidase (HRP) substrate (Millipore, Massachusetts).

#### 2.8.3 *Quantification of EV content in conditioned medium*

As a surrogate of EV quantities, protein contents were measured using a BCA protein assay kit (Thermo Scientific, 23 227). Bovine serum albumin (BSA) standards (10 μL) were added to a 96 well plate after which 200 μL of working reagent was added (50:1 ratio of reagents A and B). EV samples were diluted in CST lysis buffer (Cell Signaling Technology, 9803), vortexed, and incubated for 1 hour on ice. Ten microliters of sample lysates were added to the plate and mixed with 200 μL of working reagent. The plate was incubated for 30 minutes at 37°C and absorbance read on a spectrophotometer at 562 nm. BCA assay results combined with the volume of the isolate were used to calculate the total quantity of protein in the EV isolates and this value was used to calculate the original concentration of EV protein in the conditioned medium.

#### 2.8.4 *Particle sizalysis*

Particle size analysis was performed on EV samples using a NTA NS500 system (NanoSight, Amesbury, UK). EV samples were diluted 1:50 in PBS and injected into the NTA system, which obtained 4 × 40 second videos of the particles in motion. Videos were then analyzed with the NTA software to determine particle size.

### 2.9 Uptake of EVs by MSCs

For fluorescent labeling, 2 μg of EVs were incubated with 2 μM PKH26 dye solution (PKH26GL, Sigma) for 5 minutes, after which staining was inhibited via addition of 1% BSA solution for 1 minute. Labeled EVs were pelleted, the excess dye solution aspirated, and washed twice with culture medium. hMSCs were seeded at a density of 10 000 cells/cm^2^ in Nunc glass‐bottomed dishes (150680, Thermo Fisher) and cultured for 24 hours. Cells were washed with PBS before being incubated with either PKH26‐labeled EVs or a dye control containing no PBS with no EVs. Cells were fixed after 18 hours and stained with Alexa Fluor 488 phalloidin (1:40) (A12379, Thermo Fisher) and 4′,6‐diamidino‐2‐phenylindole (DAPI) (1:2000) (D9542, Sigma) to label the actin cytoskeleton and nuclei before mounting with Fluoroshield (F6182, Sigma) and imaged using confocal microscopy.

### 2.10 Assessing osteogenic differentiation of MSCs via ALP activity

To assess osteogenic differentiation of hMSCs, cells were seeded at a density of 2500 cells/cm^2^ in 24 well plates and cultured for 7 days with control medium containing 2% FBS and 1% PS, minimum ostegenic medium containing 10 nM dexamethasone, 0.025 mM L‐ascorbic acid and 10 mM β‐glycerol phosphate. These concentrations represent minimal levels for the support of osteogenesis.^32^, ^37^ The role of EVs was evaluated by supplementing the minimum osteogenic medium during seeding and at each medium change with EV‐S, EV depleted CM‐S, EV‐F, or EV depleted CM‐F. For EV groups, a total of 1 μg EVs were added to each well in 24‐well plates. Extracellular ALP at days 3 and 7, and intracellular ALP at day 7 were quantified using a SigmaFast p‐nitrophenyl phosphate (pNPP) kit (N1891, Sigma‐Aldrich). Standard curves were constructed using serial dilutions of p‐Nitrophenyl phosphate (pNPP, Sigma Aldrich, N1891) with 10 μL of 43 μM ALP enzyme (Sigma Aldrich, P6774) in 96‐well plates. For test samples, 50 μL of 5 mM pNPP was added to each well, with 40 μL cell lysate or cell culture medium being added followed by 40 μL ddH20. Samples were incubated for 1 hour in the dark at room temperature, after which reactions were stopped using 20 μL of 3 M NaOH and the plate was read at 405 nm. ALP activity was calculated as the amount of pNPP generated by samples, divided by sample volume and reaction time. Intracellular ALP groups were normalised to DNA content as measured using a Quant‐iT PicoGreen dsDNA Assay Kit (Invitrogen, P7589), with excitation and emission wavelengths of 485 nm and 528 nm, respectively.

### 2.11 Statistical analyses and bioinformatics

Statistical analysis on recruitment and gene expression data was carried out using one‐way analysis of variance (ANOVA) and Bonferroni's multiple comparison post‐test (*P < .05, **P < .01, ***P < .001. ^&&&^ *P* < .001 of positive control compared to all other groups).

Bioinformatic analysis was performed using Perseus 1.5.5.3^38^ to analyses LFQ data from MaxQuant. Potential contaminants, proteins identified in the decoy reverse database, and proteins identified only by site modification were omitted. LFQ values were transformed using a log2(x) function. For clustering and principal component analysis (PCA), imputation was carried out (width = 0.3, down shift = 1.8) where missing values were replaced by values from a normal distribution. For hierarchical clustering, log transformed intensities were normalized by *z*‐score and clustered using the Euclidean distance method for both columns and rows. Pathway enrichment analysis of clusters was carried out using a Fisher's exact test with the Benjamini‐Hochberg FDR threshold set to 5%, with gene ontology cellular component (GOCC), biological process (GOBP), molecular function (GOMF), and UniProt keywords being analyzed for enrichment. A Student's *t* test with a permutation‐based FDR (1, 15, 40%) was carried out to identify differences in expression of proteins between groups, and volcano plots constructed with difference (log2 fold change) on the *x*‐axis and significance (−log10 transformed) on the *y*‐axis. The difference on the *x*‐axis corresponds to the difference between the mean expression values of log2 transformed data, where a difference of n corresponds to fold change of 2n. Pathway enrichment analysis was carried out these significantly upregulated proteins using the Fisher's exact test with Benjamini‐Hochberg FDR cutoff of 5%. Results were represented as word clouds, with the size of the word representing degree of enrichment and color representing FDR corrected p value. All terms with a minimum of 0.5 enrichment factor were included. StringDB^39^ was used to generate protein‐protein interaction networks of differentially expressed proteins and perform functional enrichment analysis of gene ontology and protein family (Pfam) terms. For further analysis between CM‐S and CM‐F groups, only proteins that were identified in all three biological replicates if at least one of the groups were considered for further analysis.

## 3 RESULTS

### 3.1 Osteocytes regulate human MSC recruitment and osteogenesis in response to fluid shear

**FIGURE 1:**
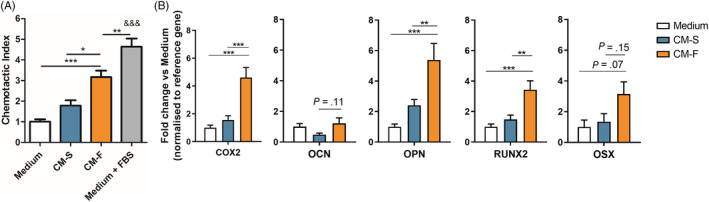
Role of osteocyte conditioned medium on hMSC osteogenesis. A, Migration of human bone marrow stem/stromal cell (hMSCs) toward osteocyte conditioned medium and normalized to Medium, showing significant increases in chemotactic index towards CM‐F medium when compared to CM‐S (n = 9). B, quantitative polymerase chain reaction (qPCR) analysis of COX‐2, OCN, OPN, RUNX2, and OSX expression in hMSCs treated with osteocyte medium from CM‐S and CM‐F (n = 4‐6). Statistical analysis using one‐way analysis of variance (ANOVA) and Bonferroni's multiple comparison post‐test for chemotactic index (*P < .05, **P < .01, ***P < .001, ^&&&^ *P* < .001 vs Medium and EV‐S)

hMSCs were cultured in conditioned medium collected from statically (CM‐S) and dynamically (CM‐F) cultured osteocytes, with recruitment and osteogenic gene expression being investigated (Figure 1A). A trend of increased hMSC recruitment toward CM‐S compared to control medium was observed; however, this was not significant. CM‐F did, however, enhance MSC recruitment; an effect that was significantly greater than with either medium (3.2‐fold; *P* < .001, n = 9) or CM‐S (1.8‐fold; *P* < .01, n = 9), indicating the enhanced chemotaxis displayed by MSCs toward mechanically stimulated osteocytes. The role of osteocyte paracrine signaling in driving osteogenesis was also investigated by treating hMSCs with CM‐S or CM‐F for 24 hours and investigating expression of osteogenic genes COX2, OCN, OPN, RUNX2, and OSX (Figure 1B). Treatment with CM‐S did not significantly alter expression of any of the investigated genes in hMSCs compared to medium. hMSCs cultured in CM‐F resulted in consistently increased expression of all genes evaluated, with significant fold changes of 4.6 in COX2, 5.4 in OPN, and 3.4 in RUNX2 compared to medium (*P* < .001, n = 4‐6). These genes were also significantly upregulated with CM‐F compared to CM‐S with 3.0‐fold, 2.2‐fold, and 2.3‐fold changes, respectively (*P* < .01‐.001, n = 4‐6). There was a near‐significant 3.1‐fold increase in OSX with CM‐F compared to medium (*P* = .07, n = 4‐6), in addition to a 2.5‐fold increase in OCN expression in CM‐F compared to CM‐S. In summary, CM‐S elicits marginal increases in hMSC osteogenesis, with significant increases following CM‐F treatment, supporting the importance of mechanical loading in mediating osteocyte‐MSC mechanosignaling.

### 3.2 Overview of identified proteins within the osteocyte secretome

**FIGURE 2:**
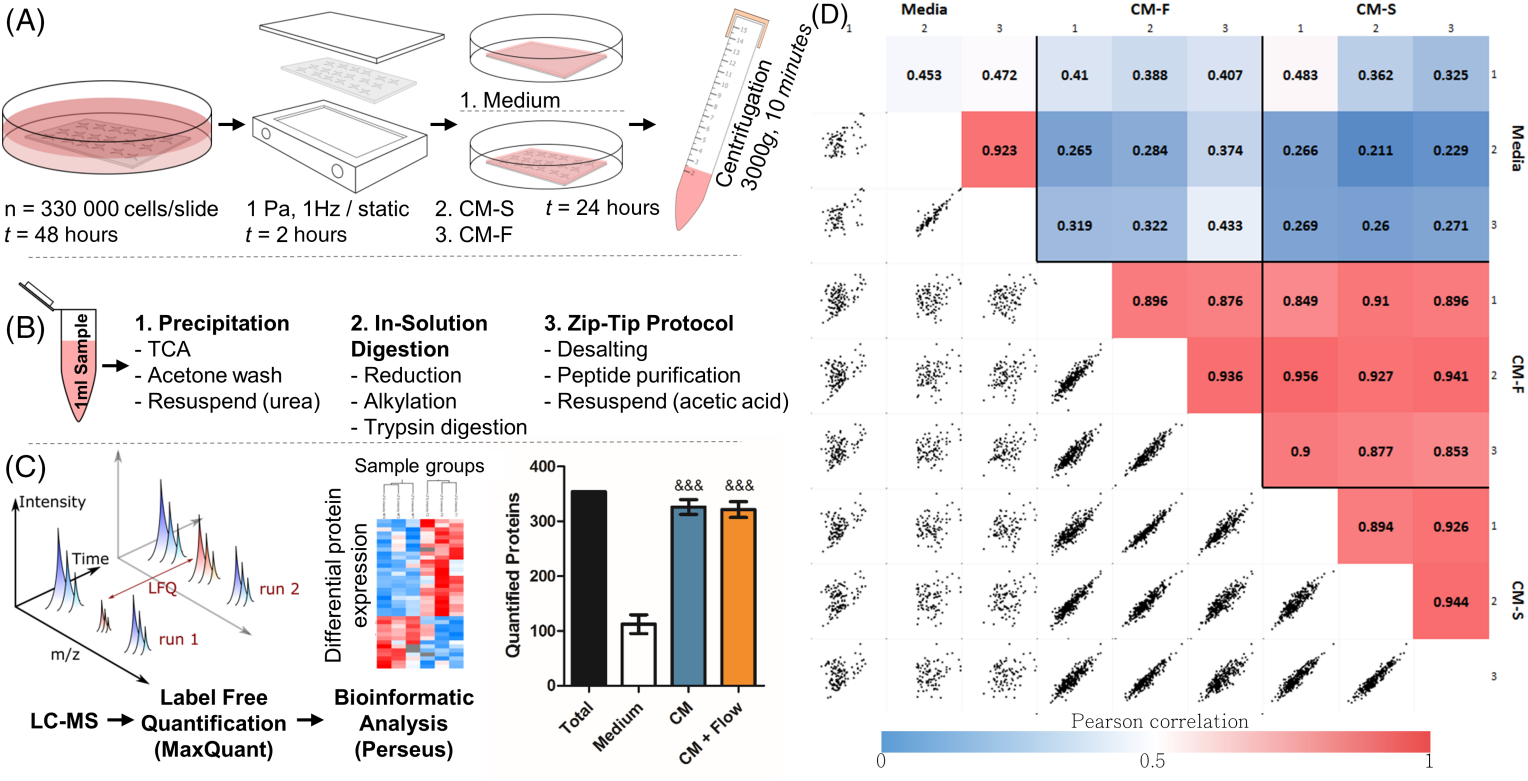
Outline of experiment procedure. MLO‐Y4 cells were seeded to collagen coated glass slides and cultured for 48 hours (A), before being transferred to parallel plate flow chambers for dynamic (OFF, 1 Pa, 1 Hz, 2 hours) or static culture. The slides were then transferred to culture dishes and 2.5 mL of serum free medium was applied, with a control group being present with collagen coated glass slides without cells. The serum‐free medium was collected and centrifuged to remove debris. One milliliter of each sample was collected, and proteins were precipitated and digested in solution before being purified via C18 stage tips (B). Samples were analyzed via liquid chromatography‐mass spectrometry and tandem mass spectrometry (LC‐MS/MS), and label‐free quantification was carried out in MaxQuant before a bioinformatic analysis was completed in Perseus (^&&&^ *P* < .001 vs Medium using one‐way analysis of variance (ANOVA) and Bonferroni's multiple comparison post‐test) (C). Pearson correlations between technical replicates, biological replicates, and sample groups were determined, with correlations between biological replicates with combined technical replicates shown (D)

Analysis of the osteocyte secretome revealed a total of 393 proteins across all groups. Within these groups, over 300 proteins were identified in both the CM‐S and CM‐F groups, with 112 being identified in Medium control (Figure 2C), with all proteins being listed in Supporting Information Table A. Pearson correlations, comparing all biological replicates to one another, show that there is a high average correlation between replicates in the CM‐S (0.92) and CM‐F (0.90) groups (Figure 2D). When comparing CM‐S and CM‐F to one another, an average correlation of 0.90 is seen, revealing a significant degree of similarity in protein expression between osteocytes cultured in static and dynamic conditions. In contrast, when comparing CM groups (CM‐S and CM‐F) to Medium, an average correlation of 0.33 was seen between them, demonstrating the difference in the osteocyte secretome and osteocyte culture media indicating the release of proteins into culture medium from the osteocyte.

### 3.3 Proteomic analysis of the osteocyte secretome reveals enrichment of proteins associated with EVs

**FIGURE 3:**
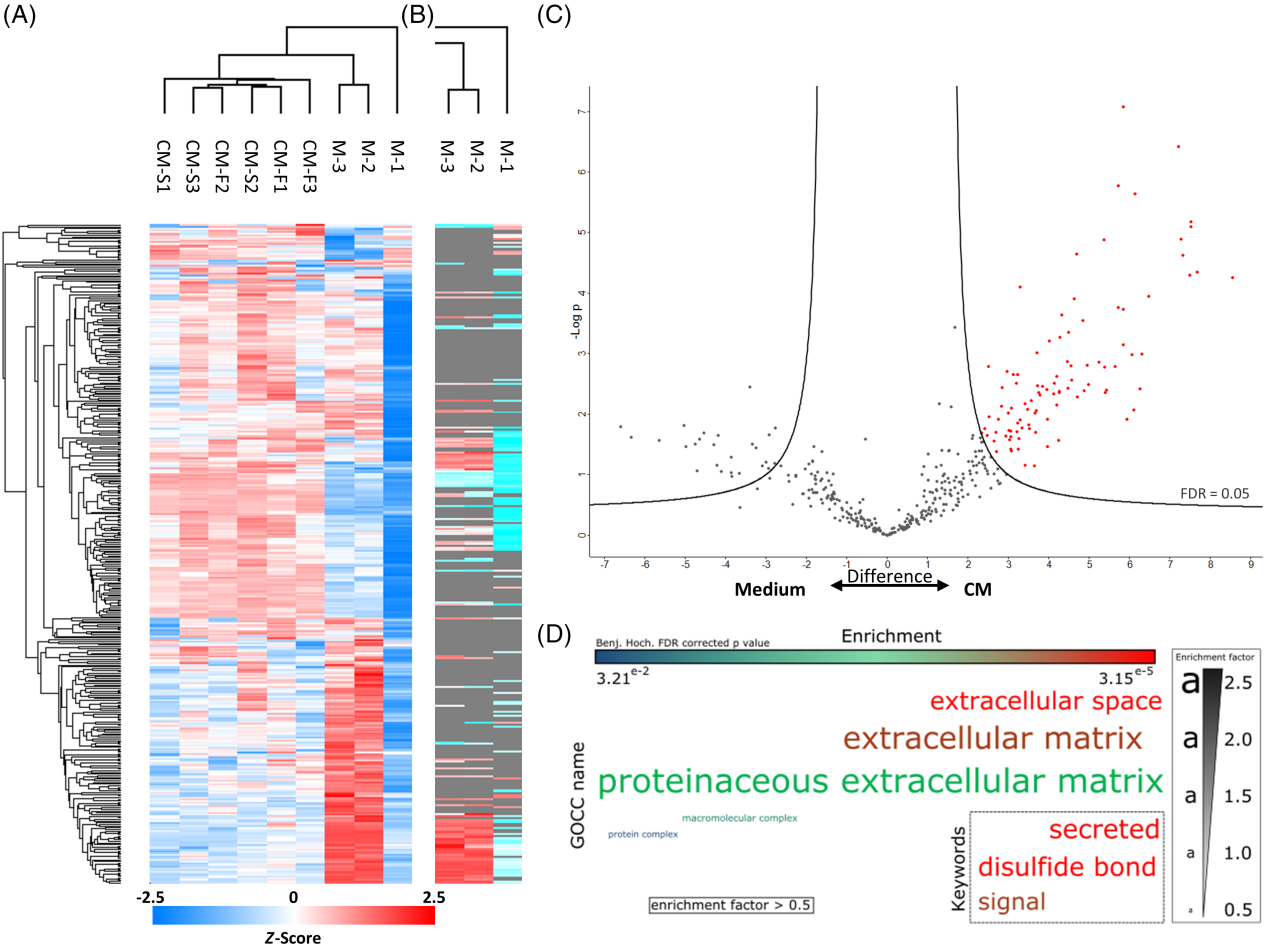
Proteomic analysis of the osteocyte secretome. Hierarchical clustering of all samples with imputed data (A) and hierarchical clustering in the control samples without imputation of data (B). Volcano plot illustrating proteins significantly upregulated proteins marked in red in CM‐S and CM‐F groups compared to the control (C). Enrichment analysis of GOCC terms and Uniprot keywords in upregulated proteins using Fisher's exact test represented as a word cloud (D). The size of the word represents enrichment of terms, while color represents FDR corrected *P* value. All terms with a minimum of 0.5 enrichment factor and 0.05 FDR corrected *P* value were included. FDR, false discovery rate

Hierarchical clustering revealed three primary groups of protein expression within the samples. CM‐S and CM‐F groups comprise one of the main clusters (Figure 3A), where it can be seen that there is considerable similarity of protein content in terms of LFQ intensity within these groups. Medium samples comprise the remaining column clusters, where the reduced number and expression of proteins are more apparent when considering data without imputation (Figure 3B). Due to the similarity between osteocyte conditioned medium groups, also verified via PCA (Figure S2), an analysis was first undertaken by combining CM‐S and CM‐F (termed CM), and comparing it to Medium to identified the proteins which comprise the osteocyte secretome. The results of this reveal the presence of 97 proteins which have significant differential expression in CM, indicated in red in Figure 3C, and listed in Table 1. Within these proteins, significant enrichment (enrichment factor > 1.7, *P* < 10^‐4^) of several “extracellular” GOCC terms was shown in comparison to the total 393 identified proteins using Fisher's exact test, with enrichment of UniProt keywords “secreted” and “signal” (enrichment factor > 1.6, *P* < 10^‐5^) also occurred (Figure 3D). This validates the successful isolation of proteins released by the osteocyte into their surrounding environment, with evidence for further downstream signaling functions. Functional enrichment within CM proteins of GOCC terms with reference to the whole *Mus musculus* genome further reported the significant enrichment of membrane‐bound vesicles and exosomes in the secretome (Table S2). This suggested a potential role for EVs, and in particular exosomes (FDR < 10^‐40^), in transporting signaling factors, either protein to RNA based, released by osteocytes. Functional enrichment of GOBP, GOMF, and Pfam terms was also investigated, showing significant roles for these proteins in mechanosensensing and mechanosignaling, as evidenced by the most significantly enriched terms “response to stress” (FDR < 10^‐6^) and “protein complex binding” (FDR < 10^‐8^). The interaction network between identified proteins in the osteocyte secretome reveals a highly significant degree of protein‐protein interaction (*P* < 10^‐16^) as illustrated in Figure S1. Enrichment analyses was also conducted on proteins more abundantly expressed in the control Medium samples using Fisher's exact test (Figure S3) and functional enrichments (Table S4), revealing enrichment of muscle and cytoskeletal terms. These associations are likely due to the incorporation of proteins from rat tail collagen type 1 used for coating glass slides.

**TABLE 1:** Differentially expressed proteins between CM‐S and CM‐F groups

Gene name	Protein	Mol. weight (kDa)	Difference (Log2 fold change)	*P* value summary
*Rpl8*	60S ribosomal protein L8	28.024	0	+
*Clic4*	Chloride intracellular channel protein 4	28.729	3.328	+
*Anxa5*	Annexin A5	35.752	2.390	**
*Ywhae*	14‐3‐3 protein epsilon	29.174	2.327	***
*Rps18*	40S ribosomal protein S18	12.483	2.177	*
*Hist2h4*	Histone H4	11.367	2.001	*
*Ywhab*	14–3‐3 protein beta/alpha	28.086	1.917	***
*Ywhaz*	14–3‐3 protein zeta/delta	27.771	1.892	**
*Dbi*	Acyl‐CoA‐binding protein	10.000	1.878	*
*Ywhag*	14–3‐3 protein gamma	28.302	1.827	**
*Try10*	MCG140784	26.221	1.762	**
*Igf2*	Insulin‐like growth factor II	11.107	1.760	*
*Hist1h2bk*	Histone H2B	13.920	1.666	
*Ptms*	Parathymosin	11.430	1.541	**
*Sh3bgrl3*	SH3 domain‐binding glutamic acid‐rich‐like protein 3	10.477	1.489	***
*Ywhaq*	14‐3‐3 protein theta	32.221	1.434	**
*Tmsb10*	Thymosin beta‐10	5.026	1.387	**
*Erh*	Enhancer of rudimentary homolog	12.259	1.126	**
*Ftl1*	Ferritin; Ferritin light chain 1; Ferritin light chain 2	20.772	1.110	**
*Inhba*	Inhibin beta A chain	47.392	0.986	**
*Myl12b*	Myosin regulatory light chain 12B	19.895	0.954	**
*Myl6*	Myosin light polypeptide 6	16.930	0.918	*
*Col1a2*	Collagen alpha‐2(I) chain	129.560	0.874	**
*Cfl1*	Cofilin‐1	18.559	0.861	**
*Gsn*	Gelsolin	85.941	−0.925	**
*Pgm1*	Phosphoglucomutase‐1	61.417	−1.135	*
*Psmd3*	26S proteasome non‐ATPase regulatory subunit 3	60.718	−1.158	***
*Aebp1*	Adipocyte enhancer‐binding protein 1	128.360	−1.319	***
*C1ra*	Complement C1r‐A subcomponent	80.072	−1.437	*
*Thbs2*	Thrombospondin‐2	129.880	−1.723	*
*Efemp1*	EGF‐containing fibulin‐like extracellular matrix protein 1	54.952	−1.732	*
*Efemp2*	EGF‐containing fibulin‐like extracellular matrix protein 2	49.425	−1.743	***
*Mdh2*	Malate dehydrogenase, mitochondrial	35.611	−2.056	*
*Npm1*	Nucleophosmin	28.385	0	+

*Note*: *P < .1, **P < .05, ***P < .01 and “+” indicates proteins with only a single or no detection in CM‐S group (top of table) and no detection in CM‐F group (bottom of table) where *P* value cannot be defined.

### 3.4 Mechanical stimulation alters the protein release characteristics in osteocytes

**FIGURE 4:**
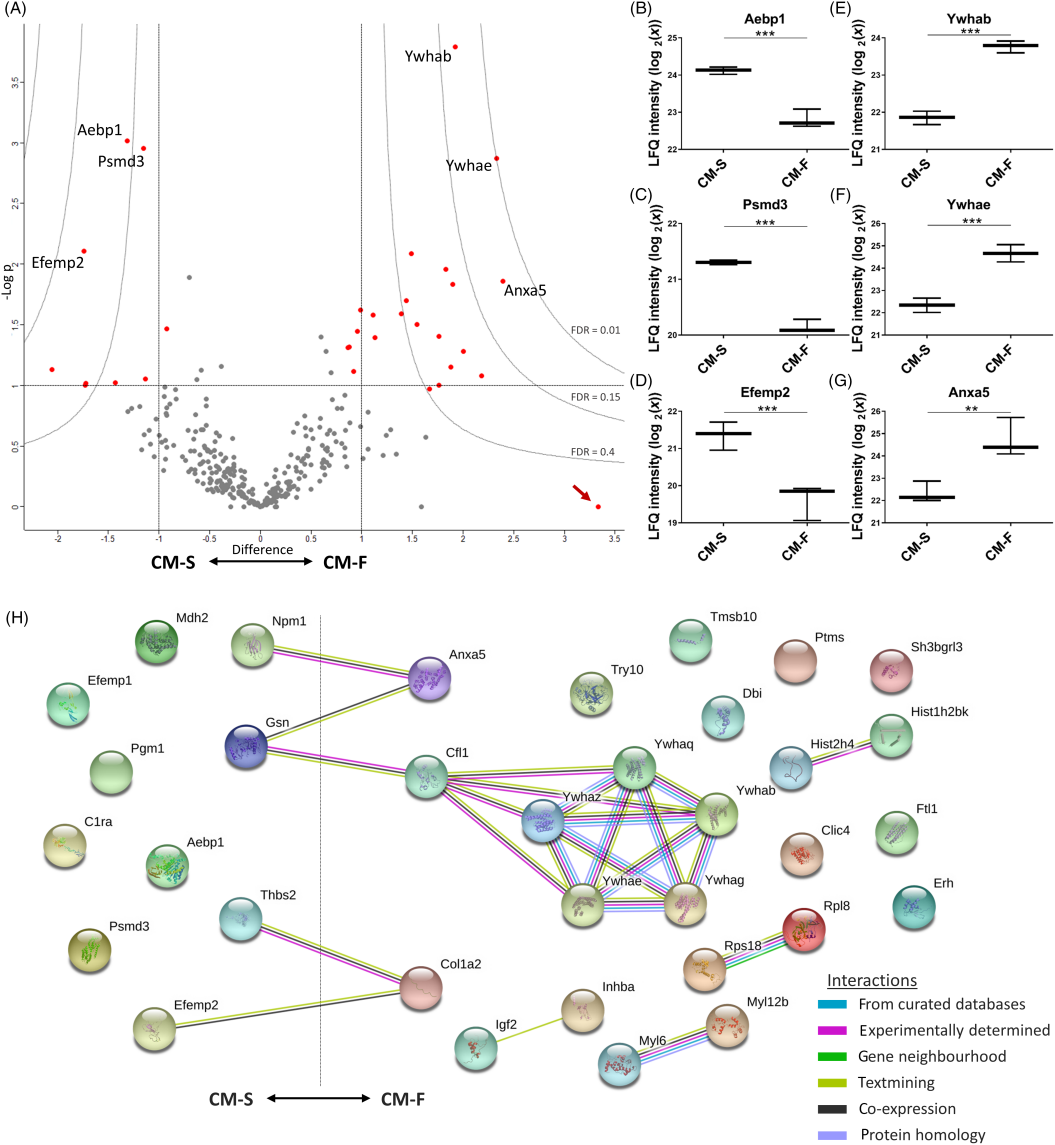
Quantifying differences between the static and dynamic osteocyte secretome. A, Volcano plot, illustrating upregulation with flow to the right and downregulation to the left. The *y*‐axis displays the ‐log10 of *P* value, where the horizontal line corresponding to a *P* value of .1. Vertical lines indicate a log2 fold change of ±1. Curves illustrate indicated FDR values with S0 parameter set to 2. B‐D, Whisker plots of three significantly upregulated proteins and E‐G significantly downregulated proteins in the presence of fluid flow are indicated. The arrow indicates a protein which displays low significance due to being present in only one of the CM replicates. H, String DB network illustrating interactions between mechanically regulated proteins, with significant degree of protein‐protein interaction (*P* < 10‐3). CM, conditioned medium; FDR, false discovery rate

Subsequent analysis separating the CM‐S and CM‐F groups showed that different proteins were released from statically cultured and mechanically stimulated osteocytes, highlighting the role of external mechanical forces in regulating the osteocyte secretome. The more stringent criteria of only considering proteins identified in all three biological replicates in at least one of the groups reduced the total number of proteins of interest to 317. A total of 34 proteins were identified with varying degrees of significance and differential expression between groups, with 32 of these indicated on a volcano plot (Figure 4A), and a further two not present on the plot due to being present in only one of the CM groups. LFQ intensities of some of the most differentially expressed proteins with greater expression in CM‐S (Figure 4B‐D) or CM‐F (Figure 4E,F) are highlighted. Of note is the enrichment of 14‐3‐3 proteins, all of which are upregulated in CM‐F (log2 fold change = 1.43‐2.33). Of particular interest are annexin A5 (log2 fold change = 2.39), which is associated with EVs and blood microparticles suggesting a role in systemic signaling, and histone H4 (log2 fold change = 2.00) which is associated with osteogenic growth peptide (OGP) and known to stimulate osteoblast activity.^40^

Subsequently, functional enrichment in differentially secreted proteins between CM‐F and CM‐S was investigated to help further elucidate their collective biological relevance in mechanically mediated osteocyte signaling (Table S3). The top four enriched GOCC terms: extracellular region, membrane‐bounded vesicle, extracellular region part, and extracellular exosome are associated with EV proteins with a highly significant FDR (<10‐10). 65% to 76% of all differentially secreted proteins were associated with these terms. This confirms that EVs are not only implicated in the osteocyte secretome, as demonstrated above, but are a key component of mechanically mediated signaling. Also, of substantial interest is the enrichment of the top two GOMF terms “calcium ion binding” (FDR < 0.01) and “phosphoserine binding” (FDR < 0.05), revealing the potential role of mechanically activated osteocyte EVs as sites of mineralization via binding of calcium and phosphate components, which has been previously postulated.^20^, ^24^ A String DB network was constructed to further investigate any potential interactions between proteins associated with EVs (Figure 4H) revealing a significant degree of protein‐protein interaction (*P* < 10‐3). Interestingly, there are several interactions between positively and negatively regulated proteins, including an interaction path between Anxa5 and Ywhab/Ywhae which are associated with calcium ion binding and phosphoserine binding, respectively. Between these nodes are gelsolin and cofilin, the former of which is calcium‐sensitive and both of which have been shown to regulate changes in the actin cytoskeleton,^41^ as well as occurring in vesicles from mineralizing osteoblasts.^42^

### 3.5 EVs are present within the osteocyte secretome and EV morphology and size distribution is not altered by mechanical stimulation

**FIGURE 5:**
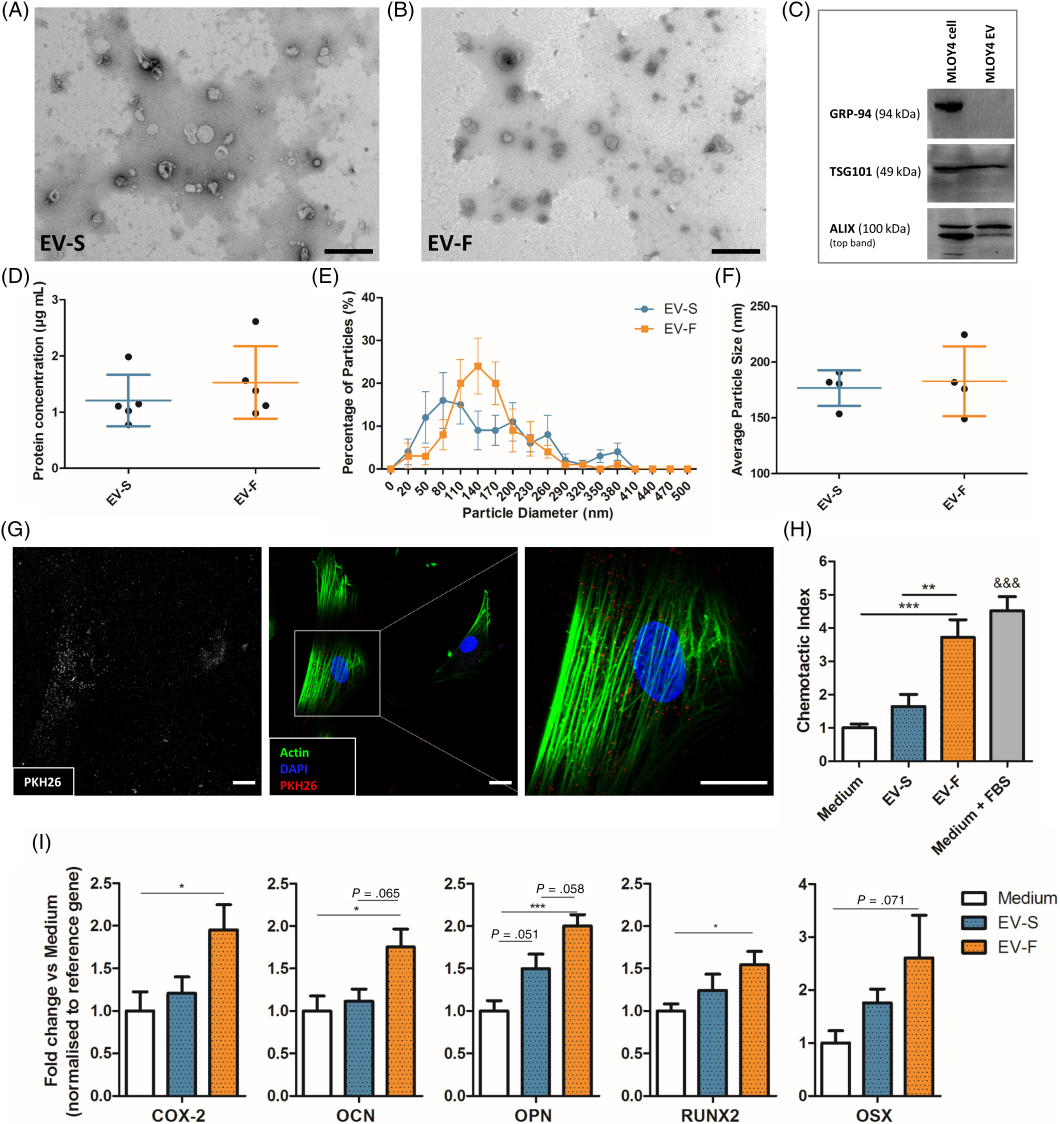
Characterization of EVs and their influence on hMSC osteogenesis. TEM image of EVs isolated from osteocyte CM‐S (A) and CM‐F (B). C, Immunoblots confirmed the presence of EVs via negative marker GRP‐94 and positive markers TSG101 and ALIX. D, Protein concentration of EVs in conditioned medium groups (n = 5). Nanoparticle size analysis on EVs confirmed no significant difference in distribution (E) or average size (F) between groups (n = 4). G, Immunofluorescent images illustrating osteocyte EV uptake by human bone marrow stem/stromal cell (hMSCs), as demonstrated by localization of PKH26 labeled EVs within the cell body (scale bar = 10 μm). H, Migration of hMSCs toward EVs isolated from osteocyte conditioned medium and normalized to Medium, showing significant increases in chemotactic index toward CM‐F medium when compared to CM‐S. I, Quantitative polymerase chain reaction (qPCR) analysis of COX‐2, OCN, OPN, RUNX2, and OSX expression in hSSCs treated with EVs from osteocyte medium from CM‐S and CM‐F. Statistical analysis using one‐way analysis of variance (ANOVA) and Bonferroni's multiple comparison post‐test (*P < .05, **P < .01, ***P < .001, ^&&&^ *P* < .001 vs Medium and EV‐S). EVs, extracellular vesicles; TEM, transmission electron microscope

Given the identification of EV‐associated proteins within the osteocyte secretome, we next investigated whether osteocytes release EVs and, if so, whether EV characteristics were altered by mechanical stimulation. EVs were successfully separated from osteocyte CM using filtration and ultracentrifugation, with the presence of EVs confirmed by TEM imaging and immunoblotting. TEM imaging confirmed the presence of EVs of typical morphology and size (Figure 5A,B). The presence of EVs was further confirmed via immunoblotting, with no detection of negative marker GRP‐94, and detection of positive markers TSG101 and ALIX (Figure 5C). EV concentration was not significantly different between EVs separated from the CM‐S (EV‐S) and EVs separated from the CM‐F (EV‐F), both being within the range of 0.8‐2.6 μg/mL, and with average values of 1.2 μg/mL and 1.5 μg/mL, respectively (Figure 5D). It can be seen that there is a change in particle size distributions between EV‐S and EV‐F (Figure 5E); however, no changes in average particle size was detected, with values of 177 nm and 183 nm, respectively (Figure 5F).

### 3.6 Osteocytes regulate human MSC recruitment and osteogenesis in response to fluid flow shear via MA‐EVs

To determine whether murine osteocyte‐derived EVs could be taken up by human MSCs, we labeled EVs with PKH26. Following 24 hours treatment, labeled‐EVs were preferentially located within the cytoplasm, indicating uptake of EVs by hMSCs (Figure 5G). Control samples are illustrated in Figure S4. A high density of EVs can be seen around the nuclear region in particular with minimal detection within the nucleus.

Upon verifying EV uptake, the cellular response of hMSCs subjected to EVs separated from CM‐S (ie, EV‐S) or CM‐F (EV‐F) was investigated. Specifically, hMSCs were treated with EV‐S and EV‐F to investigate recruitment and osteogenic gene expression as previously demonstrated with CM. EV‐S resulted in a slight nonsignificant increase in MSC recruitment (Figure 5H), while this response was significantly enhanced with EV‐F, yielding a 3.7‐fold increase compared to medium (*P* < .001, n = 9) and 2.3‐fold increase compared to EV‐S (*P* < .01, n = 9). This trend closely mirrored that seen with whole secretome. Osteogenic gene expression (Figure 5I) showed a consistent trend of marginally increased expression with CM‐S treatment, which was further enhanced with CM‐F. There was a near‐significant increase of 1.5‐fold in OPN (*P* = .051, n = 17‐18) when comparing CM‐S to Medium. CM‐F resulted in significant changes compared to medium of 2.0‐fold in COX2 (*P* < .05, n = 17‐18), 1.8‐fold in OCN (*P* < .05, n = 14‐15), 2.0‐fold in OPN (*P* < .001, n = 16‐18) and 1.5‐fold in RUNX2 (*P* < .05, n = 20), with a near‐significant increase of 2.6‐fold in OSX (*P* = .07, n = 21‐23). In addition, near‐significant increases in OCN and OPN were detected comparing EV‐F and EV‐S. Moreover, orthology searches using the basic local alignment search tool BLAST^43^ were performed for the human gene sequences (COX2, OCN, OPN, RUNX2, OSX) in the murine genome (version: *Mus musculus* GRCm38.p4). The bioinformatic tool, in silico PCR (UCSC Genome Browser), was used to confirm the lack of amplification of the human primer sequences in the murine genome,^44^ confirming that amplified genes are human, and not due to possible transfer of murine mRNA from the MLO‐Y4 cell line. In summary, there is a trend of increasing osteogenesis in hMSC following EV‐S treatment. However, this affect becomes significantly greater with EV‐F treatment, showing a similar trend to that seen with whole secretome and demonstrates that EVs from mechanically‐activated osteocytes are key drivers of stem cell recruitment and osteogenesis.

**FIGURE 6:**
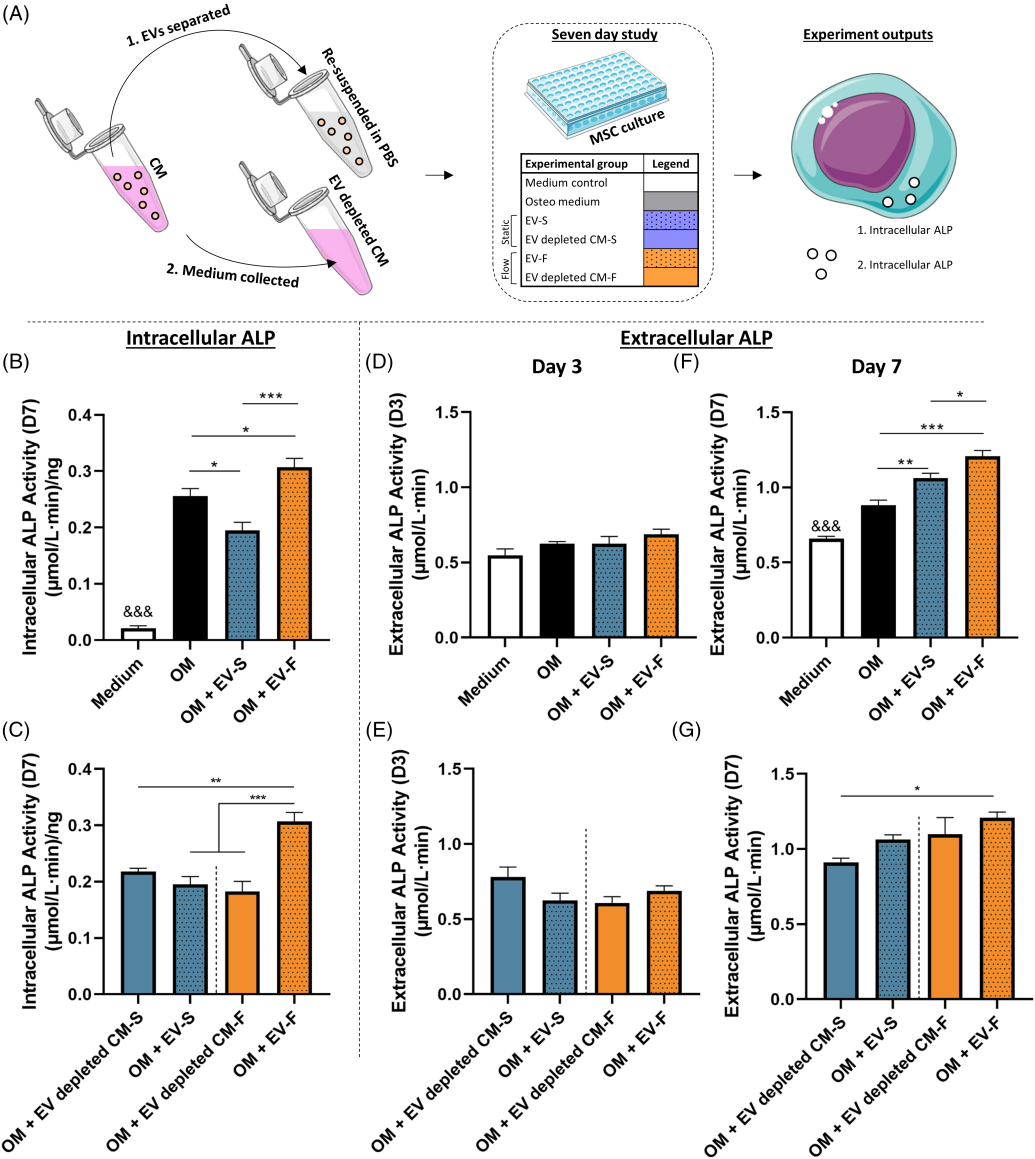
Long‐term study of influence of EVs on human bone marrow stem/stromal cell (hMSC) osteogenesis. A, Summary schematic of experimental procedure and outputs. Intracellular ALP activity at day 7 (B), with a comparison of EV groups with EV depleted conditioned medium (C). Extracellular ALP activity at day 3 (D) with a comparison of EV groups with EV depleted conditioned medium (E). Extracellular ALP activity at day 7 (F) with a comparison of EV groups with EV depleted conditioned medium (G). All data n = 6. Statistical analysis using one‐way analysis of variance (ANOVA) and Tukey's multiple comparison post‐test (*P < .05, **P < .01, ***P < .001, ^&&&^ *P* < .001 of indicated group vs all other groups). ALP, alkaline phosphatase; EVs, extracellular vesicles

To further investigate the role of EVs in later stage differentiation, hMSCs were cultured up to 7 days, with intracellular ALP being investigated at day 7 and extracellular ALP being investigated at day 3 and day 7. This was conducted on both EVs and EV depleted medium to investigate how other non‐EV associated factors within the medium affect osteogenesis (Figure 6A). Intracellular ALP activity was significantly enhanced at day 7 with EV‐F vs EV‐S in addition to all other groups (Figure 6B), while it was also demonstrated that EV‐F significantly enhanced ALP activity compared to both static and flow EV depleted medium (Figure 6C), which is consistent with trends seen with earlier gene expression. Extracellular ALP was also assessed from the medium change at day 3, in addition to the end of the experiment at day 7. There were no significant differences between any groups at day 3 (Figure 6D,E). However, at day 7 it can be seen that EV‐F is significantly enhanced compared to EV‐S (Figure 6F). Interestingly, while there is an increase in extracellular ALP in the EV‐F compared to the EV depleted CM‐F, this is not significant (Figure 6G). Taken together, this data demonstrate that EVs isolated from mechanically activated osteocytes can enhance the osteogenesis of hMSCs.

## 4 DISCUSSION

Osteocytes are mechanosensitive cells which play a fundamental role in coordinating loading‐induced bone formation via the secretion of paracrine factors which drive effector cell behavior. One of the most important of which are bone marrow stem/stromal cells (MSCs) which are responsible for replenishing the bone forming osteoblast population. However, the exact mechanisms by which osteocytes relay mechanical signals to these cells are poorly understood. A greater understanding of these mechanisms would thus have profound implications for the development of therapies to treat the wide range of diseases with which the osteocyte has been linked, one of the most devastating of which is osteoporosis. Therefore, this study aimed to demonstrate the potency of the mechanically stimulated osteocyte secretome in driving human MSC behavior, and fully characterize its protein content with the aim of identifying the key secreted factors regulating bone mechanobiology. Herein, we demonstrate that osteocytes subjected to oscillatory fluid shear secrete factors that significantly enhance hMSC recruitment and osteogenesis. To uncover the osteocyte derived secreted factors which drive hMSC behavior, we performed a proteomic analysis of the osteocyte secretome to uncover an extensive map of proteins which are released both under static conditions and following mechanical stimulation. Over 300 proteins comprising the osteocyte secretome were identified with 34 proteins differentially expressed following mechanical stimulation. The osteocyte secretome was significantly enriched with proteins associated with EVs and exosomes indicating a role for secreted vesicles in mediated mechanically driven osteocyte‐MSC communication. EVs were subsequently separated from the mechanical activated osteocyte secretome, characterized, and found to elicit similar trends in MSC recruitment and osteogenesis to that seen with conditioned media, demonstrating a key role for osteocyte EVs in mediating hMSC behavior.

Mechanically stimulated osteocytes secrete paracrine factors that recruit human MSCs and enhance osteogenesis. The ability of mechanically stimulated osteocytes to influence stem cell behavior is in agreement with previous findings in vivo where mechanical loading of bone results in the recruitment and osteogenic differentiation of endogenous^45^ or transplanted exogenous osteoprogenitors.^46^ Furthermore, a similar trend in recruitment has been shown in murine MSCs where a 128% increase in stem cell recruitment was observed following exposure to conditioned media collected from osteocytes cultured on a rocking platform.^10^ Interestingly in the same study, mechanically activated osteocyte conditioned media was also been shown to induce osteogenesis of MSCs as demonstrated by upregulation of Opn and Cox‐2 gene expression and enhanced mineral deposition.^10^, ^11^ We have demonstrated a comparable increase in COX‐2 and OPN expression in human MSCs, as well as increases in OCN, OSX and RUNX2. These findings, along with other previous work investigating the effect of the osteocyte secretome on osteoblast proliferation, migration and osteogenesis,^9^, ^47^ further reinforce the importance of the osteocyte secretome and its contents in the indirect biophysical regulation of MSCs and loading‐induced bone formation.^48^, ^49^

To determine the mechanisms by which osteocytes coordinate stem cell recruitment and osteogenesis in response to loading, for the first time, we identified a detailed a map of the osteocyte secretome via a mass spectrometry based proteomic analysis. Interestingly, a number of key proteins, such as Sclerostin, which is known to be secreted by osteocytes, were not detected. This may due to the limitation of the MLO‐Y4 osteocyte cell line but given the pro‐osteogenic effect of this mechanically activated osteocyte secretome, this opens the possibility of identifying novel factors regulating MSC behavior. We have identified several proteins from a previous proteomic analysis on osteocyte lysates,^18^ revealing potential roles for these proteins in cell signaling. Further analysis sought to investigate the role of mechanical forces on the contents of the osteocyte secretome, with differential expression of a range of proteins including the 14‐3‐3 protein group. Previous studies have implicated these proteins in osteogenesis, with downregulation of 14‐3‐3 beta being demonstrated in calvaria organ cultures resulting in increased bone formation^50^ and increased 14‐3‐3 epsilon release being demonstrated in osteoblasts/osteocytes in response to dynamic compression.^51^ Other proteins of particular interest are histone H4 and annexin A5. Histone H4 has been shown to play a role in osteoblast gene expression and osteogenesis^52^, ^53^ while the C‐terminus of histone H4, termed OGP plays key roles in regulating the behavior of bone residing cells.^40^, ^54^ Annexin A5 has been shown to increase at the cell membrane in addition to Ca2+ levels in osteoblasts under fluid flow. The disruption of annexin A5 inhibits Ca2+, implicating its role in calcium signaling,^55^ with its knockdown impairing osteoblast function.^56^ One downregulated protein of interest which we have identified is thrombospondin 2. The knockdown of this protein in mice has been shown to increase angiogenesis^57^ and endosteal bone formation^58^ while thrombospondin 2 null mice demonstrate enhanced callus bone formation, vascularity and MSC proliferation following tibial fracture.^59^ In addition to the above, many of the proteins we have identified are known to be EV cargo, with many also having previously been identified in osteoblast EVs.^42^, ^60^ The content of the osteocyte secretome, in addition to a possible delivery mechanism via EVs, provides us with a database of information via which to develop targeted therapeutics for specific bone diseases such as osteoporosis.

The role of EVs in osteocyte signaling to MSCs was investigated to further investigate the mechanisms behind our previously discussed findings. EVs were separated from osteocyte conditioned media, where interestingly, we did not detect any changes in EV morphology or quantity between static and dynamic groups. This is in contrast to previous work which has demonstrated an upregulation in EV number following fluid shear stimulation.^61^ We have however demonstrated an almost identical trend in stem cell recruitment with CM and EVs. Stem cell recruitment is enhanced both with medium from mechanically stimulated osteocytes, and EVs separated from the same medium, providing evidence for the key role of osteocyte derived EVs in mediating this response. Similarly, the almost identical trends in MSC osteogenic gene expression treated with CM and EVs further provide evidence for the role of osteocyte derived MA‐EVs in facilitating cell‐cell communication in bone. We have also demonstrated further long‐term effects of MA‐EVs on hMSC osteogenesis, as seen by the enhanced intracellular and extracellular ALP activity at day 7 compared to EVs secreted from statically cultured osteocytes. Given the similar concentrations of EVs between groups, it is expected that this pro‐osteogenic effect is a result of EV content changing in response to mechanical stimulation. EVs are also known to act as sites of mineral nucleation, as has been demonstrated in osteoblasts.^20^, ^62^ We have also seen evidence for this, with enrichment of calcium ion binding (such as annexin A5) and phosphoserine binding (such as 14‐3‐3 proteins Ywhae and Ywhab) proteins in our proteomic analysis. In our protein interaction network, Annexin A5 is linked to the calcium sensitive protein gelsolin,^41^ which in turn is linked to the 14‐3‐3 proteins via the phosphate regulating cofilin.^63^ In addition to the known role of calcium ions in mineralization, negatively charged amino acids such as phosphoserine are also known to play a key role in hydroxyapatite nucleation and growth.^64^ Therefore, osteocyte EVs may promote mineralization via delivery of calcium and phosphate interacting proteins through interaction with gelsolin and cofilin, respectively. Another likely mechanisms in the pro‐osteogenic capabilities of EVs are RNAs, specifically miRNAs, with a previous study demonstrating altered miRNA expression in EVs isolated from the plasma of osteocyte ablated and wild‐type mice.^25^ The potency of EVs in mediating cell behavior has led others to exploit them for the development of therapeutics. For example, EVs have been investigated as a potential therapy for root canal treatments,^65^ for the functionalization of TE scaffolds to enhance bone regeneration,^66^, ^67^ and as delivery vehicles for the loading of drugs for osteoporosis therapies.^28^ Taken together, we have shown that MA‐EVs are a key mechanism by which osteocyte communicate chemotactic and osteogenic signals to osteoprogenitors in response to loading, and present themselves as a potential cell free therapy to mimic the beneficial effect of loading and enhance bone formation.

In summary, this study presents evidence that the mechanically stimulated osteocyte secretes factors which coordinates MSC recruitment and osteogenesis demonstrating a mechanism required for loading‐induced bone formation. Importantly, for the first time, we have mapped the osteocyte protein secretome and determined how this is altered in response to mechanical stimulation generating a database of potential factors mediating this mechanism. Last, this study also demonstrates the presence and fundamental role of MA‐EVs released by osteocytes in coordinating MSC recruitment and osteogenesis, identifying a novel mechanism by which osteocytes coordinate bone mechanobiology. Moreover, these pro‐osteogenic osteocyte derived MA‐EVs represent a potential cell‐free therapy to enhance bone regeneration and repair in diseases such as osteoporosis.

## CONFLICT OF INTEREST

K.F.E. declared employment at Trinity College Dublin and research funding from Irish Research Council, European Research Council, and Science Foundation Ireland. The other authors declared no potential conflicts of interest.

## AUTHOR CONTRIBUTIONS

K.F.E.: conception and design, collection and/or assembly of data, data analysis and interpretation, manuscript writing; I.W., M.R., G.P.J., N.S., M.C., M.‐N.L.: collection and/or assembly of data, data analysis and interpretation; K.W.: collection and/or assembly of data and data analysis; M.C.L.: collection and/or assembly of data; L.O.: data analysis and interpretation, manuscript writing, financial support; D.A.H.: conception and design, data analysis and interpretation, manuscript writing, financial support, final approval of manuscript.

## Supporting information


**Appendix** **S1.** Supporting Information.

**Appendix** **S2.** Supporting Information.

## Data Availability

The data that support the findings of this study are available from the corresponding author upon reasonable request.
