# Supplementary material for: Human bone marrow stem/stromal cell osteogenesis is regulated via mechanically activated osteocyte‐derived extracellular vesicles
Source: Stem Cells Transl Med. 2020 Jul 16;9(11):1431–47. doi: 10.1002/sctm.19-0405 (PMC7581449; doi:10.1002/sctm.19-0405)
Supplement: Supplementary file 1 — Appendix S1. Supporting Information. [file SCT3-9-1431-s001.pdf]

Supplemental Files

Human bone marrow stem/stromal cell osteogenesis is regulated via mechanically activated osteocyte-derived extracellular vesicles

Authors

Kian F. Eichholz.[1,2,3], Ian Woods[1,2,3], Mathieu Riffault[1,2,3], Gillian P. Johnson[1,2,3], Michele Corrigan[1,2,3], Michelle C. Lowry[6], Nian Shen[1,2,3], Marie-Noelle Labour[1,2,3], Kieran Wynne[4,5], Lorraine O'Driscoll[6], David A. Hoey[1,2,3,7]

Affiliations

[1]Dept. Mechanical, Aeronautical and Biomedical Engineering, Materials and Surface Science Institute, University of Limerick, Limerick, Ireland

[2]Trinity Centre for Biomedical Engineering, Trinity Biomedical Sciences Institute, Trinity College Dublin, Ireland

[3]Dept. of Mechanical and Manufacturing Engineering, School of Engineering, Trinity College Dublin, Ireland.

[4]UCD Conway Institute of Biomolecular and Biomedical Research, University College Dublin, Dublin 4, Ireland.

[5]Mass Spectrometry Resource, University College Dublin, Dublin 4, Ireland.

[6]School of Pharmacy and Pharmaceutical Sciences and Trinity Biomedical Sciences Institute, Trinity College Dublin, Dublin, Ireland.

[7]Advanced Materials and Bioengineering Research Centre, Trinity College Dublin & RCSI.

Address for correspondence:

David Hoey,

Department of Mechanical and Manufacturing Engineering, School of Engineering,

Trinity College Dublin, Dublin 2, Ireland

dahoey@tcd.ie

Author Contributions:

K.F.E.: Conception and design, Collection and/or assembly of data, Data analysis and interpretation, Manuscript writing. I.W.: Collection and/or assembly of data, Data analysis and interpretation. G.P.J.: Collection and/or assembly of data, Data analysis and interpretation. N.S.: Collection and/or assembly of

data, Data analysis and interpretation. M.C.: Collection and/or assembly of data, Data analysis and interpretation. M.N.L.: Collection and/or assembly of data, Data analysis and interpretation. K.W.: Collection and/or assembly of data and Data analysis. M.C.L.: Collection and/or assembly of data. L.O'D.: Data analysis and interpretation, Manuscript writing, Financial support. D.A.H.: Conception and design, Data analysis and interpretation, Manuscript writing, Financial support, Final approval of manuscript.

Supplementary figures and tables

Table S1 Primer sequences and concentrations employed in quantitative PCR analysis.

Gene symbol	Tm (°C)	Primer concentration	Sequence	Amplicon size
18s	60	300 nM	ATCGGGGATTGCAATTATTC<br>CTCACTAAACCATCCAATCG	130bp
GAPDH	60	300 nM	ACAGTTGCCATGTAGACC<br>TTTTTGGTTGAGCACAGG	95bp
COX2	60	400 nM	AAGCAGGCTAATACTGATAGG<br>TGTTGAAAAGTAGTTCTGGG	113bp
OCN	65	400 nM	CACTCCTCGCCCTATTGGC<br>CCCTCCTGCTTGGACACAAAG	112bp
OPN	60	400 nM	GACCAAGGAAAACTCACTAC<br>CTGTTTAACTGGTATGGCAC	84bp
RUNX2	60	400 nM	GCAGTATTTACAACAGAGGG<br>TCCCAAAAGAAGTTTTGCTG	112bp
OSX	60	400 nM	TGAGGAGGAAGTTCACTATG<br>CATTAGTGCTTGTAAAGGGG	200bp

Table S2 Functional enrichments in CM proteins indicated in Figure 3C using String DB, with observed gene count out of a total of 105 genes and FDR cut-off of 2%. (note: 105 genes were identified from 97 proteins)

Pathway description	Observed gene count	False discovery rate (FDR)
GOCC (Gene Ontology Cellular Component)		
extracellular exosome	71	1.72E-41
extracellular region part	76	5.05E-39
extracellular region	77	1.20E-35
membrane-bounded vesicle	70	2.07E-34
extracellular space	47	7.88E-32
extracellular matrix	26	9.57E-22
proteinaceous extracellular matrix	22	5.85E-18
cytoplasmic membrane-bounded vesicle	26	1.80E-12
cytoplasmic vesicle	24	9.53E-10
myelin sheath	12	9.48E-09
GOBP (Gene Ontology Biological Process)		
response to stress	36	6.92E-07
antigen processing and presentation of peptide antigen via MHC class I	6	4.09E-06
protein folding	10	8.52E-06
response to wounding	13	2.05E-05
regulation of biological process	63	5.41E-05
pyruvate metabolic process	6	1.73E-04
extracellular matrix organization	9	1.73E-04
biological regulation	63	1.99E-04
glycolytic process	5	2.85E-04
response to endogenous stimulus	19	2.85E-04
GOMF (Gene Ontology Molecular Function)		
protein complex binding	20	7.80E-09
protein binding	52	7.85E-09
binding	73	5.31E-08
RNA binding	26	3.35E-07
poly(A) RNA binding	23	3.35E-07
isomerase activity	9	9.02E-06
macromolecular complex binding	21	1.05E-05

receptor binding	20	1.22E-05
calcium ion binding	15	1.22E-05
peptide binding	9	1.90E-05

Table S3 Functional enrichments in network using String DB with observed gene count out of a total of 34 genes and FDR cut-off of 2%.

Pathway description	Observed gene count	False discovery rate (FDR)	Associated proteins in network
GOCC (Gene Ontology Cellular Component)			
extracellular region	26	1.16E-11	Aebp1,Anxa5,C1ra,Cfl1,Clic4,Col1a2,Dbi,Efemp1,Efemp2,Ftl1, Gsn,Hist1h2bk,Hist2h4,Igf2,Inhba,Mdh2,Myl12b,Myl6,Pgm1,Psmd3,Sh3bgrl3,Thbs2,Ywhab,Ywhae,Ywhag,Ywhaz
membrane-bounded vesicle	24	1.16E-11	Aebp1,Anxa5,C1ra,Cfl1,Clic4,Col1a2,Dbi,Efemp1,Efemp2,Ftl1, Gsn,Hist2h4,Igf2,Mdh2,Myl12b,Myl6,Pgm1,Psmd3,Sh3bgrl3,Thbs2,Ywhab,Ywhae,Ywhag,Ywhaz
extracellular region part	25	1.16E-11	Aebp1,Anxa5,C1ra,Cfl1,Clic4,Col1a2,Dbi,Efemp1,Efemp2,Gsn, Hist1h2bk,Hist2h4,Igf2,Inhba,Mdh2,Myl12b,Myl6,Pgm1,Psmd3,Sh3bgrl3,Thbs2,Ywhab,Ywhae,Ywhag,Ywhaz
extracellular exosome	22	1.16E-11	Aebp1,Anxa5,C1ra,Cfl1,Clic4,Col1a2,Dbi,Efemp1,Efemp2,Gsn, Hist2h4,Igf2,Mdh2,Myl12b,Myl6,Pgm1,Psmd3,Sh3bgrl3,Ywhab,Ywhae,Ywhag,Ywhaz
focal adhesion	9	2.88E-07	Anxa5,Cfl1,Gsn,Npm1,Rpl8,Ywhab,Ywhae,Ywhag,Ywhaz
extracellular space	12	3.20E-06	Aebp1,Anxa5,C1ra,Cfl1,Col1a2,Dbi,Efemp1,Gsn, Hist1h2bk,Igf2,

			Inhba,Ywhaz
cytosol	13	2.65E-05	Cfl1,Clic4,Gsn,Npm1,Pgm1,Ptms,Rpl8,Rps18,Ywhab,Ywhae, Ywhag,Ywhaq,Ywhaz
cytoplasmic vesicle part	7	1.04E-04	Clic4,Ftl1,Ywhab,Ywhae,Ywhag,Ywhaq,Ywhaz
cytoplasmic membrane-bounded vesicle	9	3.24E-04	Clic4,Dbi,Ftl1,Thbs2,Ywhab,Ywhae,Ywhag,Ywhaq,Ywhaz
cytoplasmic vesicle membrane	6	5.14E-04	Clic4,Ywhab,Ywhae,Ywhag,Ywhaq,Ywhaz
blood microparticle	4	6.77E-04	Anxa5,C1ra,Gsn,Ywhaz
cell junction	9	1.06E-03	Cfl1,Clic4,Gsn,Npm1,Rpl8,Ywhab,Ywhae,Ywhag,Ywhaz
macromolecular complex	16	4.47E-03	Clic4,Col1a2,Ftl1,Gsn,Hist1h2bk,Hist2h4,Inhba,Myl12b,Myl6, Npm1,Psmd3,Rps18,Ywhab,Ywhae,Ywhaq,Ywhaz
extracellular matrix	5	1.21E-02	Aebp1,Col1a2,Efemp1,Efemp2,Thbs2
protein complex	14	1.21E-02	Clic4,Col1a2,Ftl1,Gsn,Hist1h2bk,Hist2h4,Inhba,Myl12b,Myl6, Psmd3,Ywhab,Ywhae,Ywhaq,Ywhaz
GOBP			
regulation of biological quality	14	1.60E-02	Anxa5,Cfl1,Clic4,Col1a2,Dbi,Ftl1,Inhba,Myl12b,Thbs2,Tmsb10, Ywhab,Ywhae,Ywhag,Ywhaz
GOMF (Gene Ontology Molecular Function)			
calcium ion binding	8	4.03E-03	Anxa5,C1ra,Efemp1,Efemp2,Gsn,Myl12b,Myl6,Thbs2
phosphoserine binding	2	2.65E-02	Ywhab,Ywhae
rRNA binding	3	4.06E-02	Npm1,Rpl8,Rps18
protein domain specific binding	6	4.06E-02	Gsn,Ywhab,Ywhae,Ywhag,Ywhaq,Ywhaz
Pfam			
14-3-3 protein	4	5.84E-08	Ywhab,Ywhae,Ywhag,Ywhaq

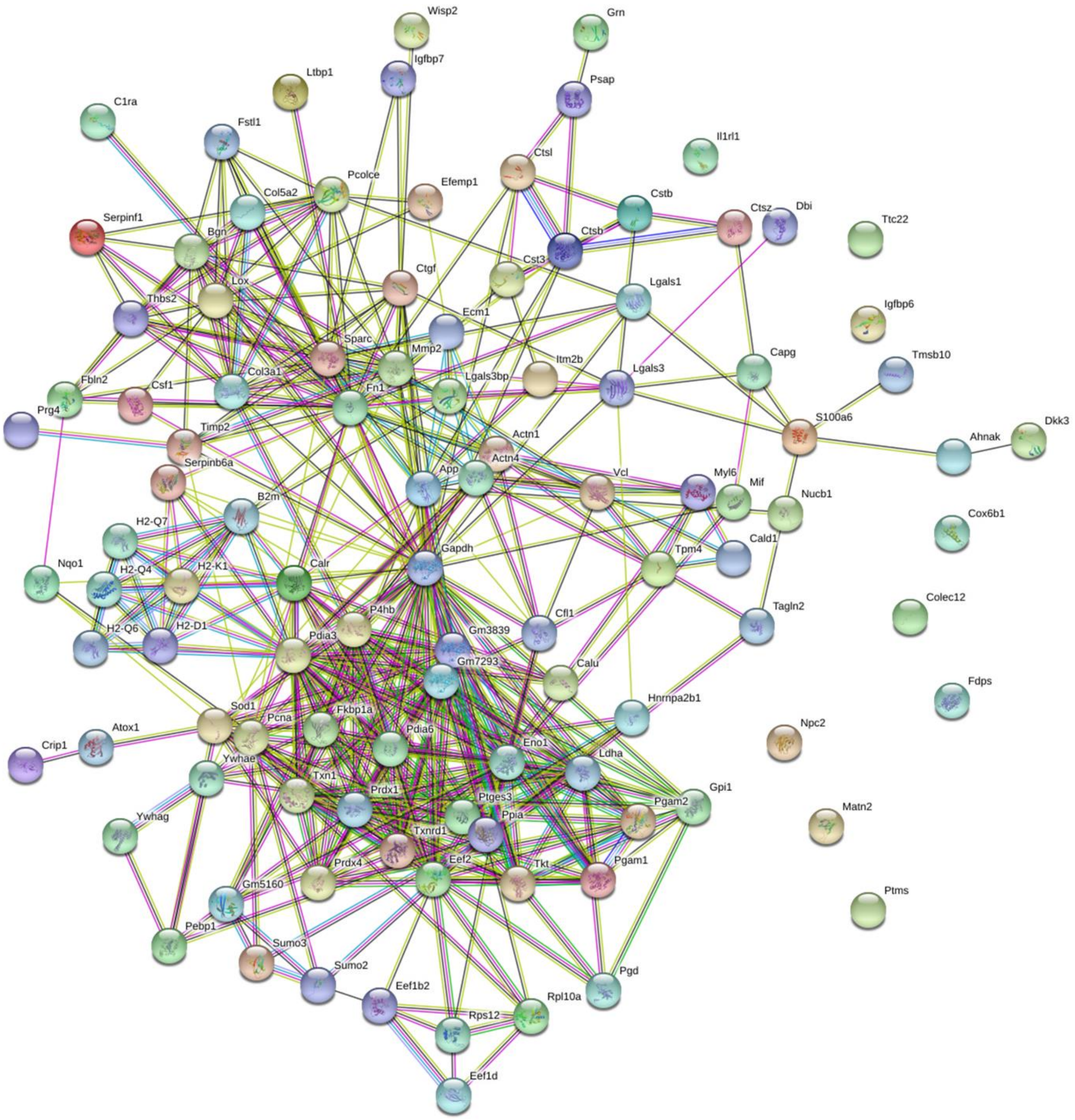

Figure S1 String DB network illustrating interactions between proteins in the osteocyte secretome, with significant degree of protein-protein interaction (p < 10-16)

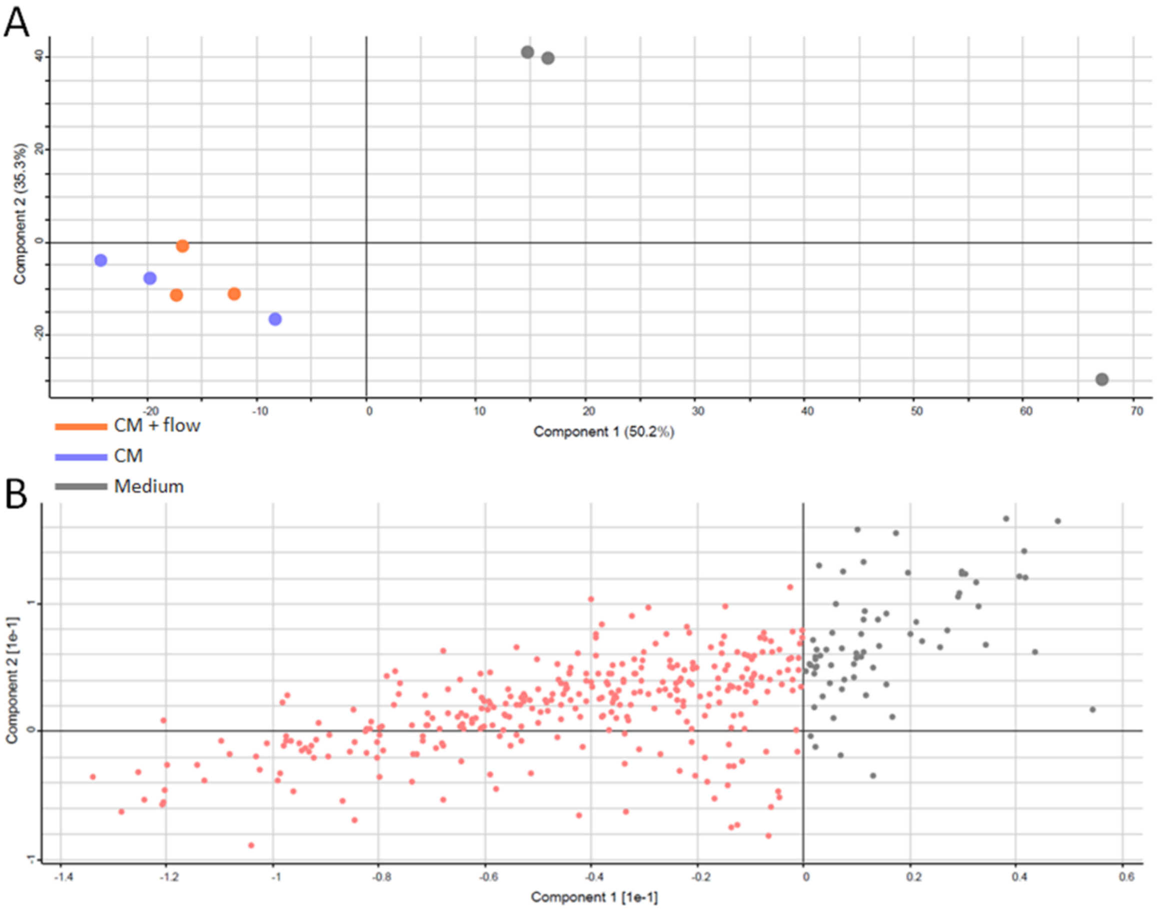

Figure S2 Principal Component Analysis (PCA) revealing the variance between the three experimental groups and indicating three main clusters of data (C). The proteins primarily driving the separation between the medium groups and the control groups are highlighted in red (D).

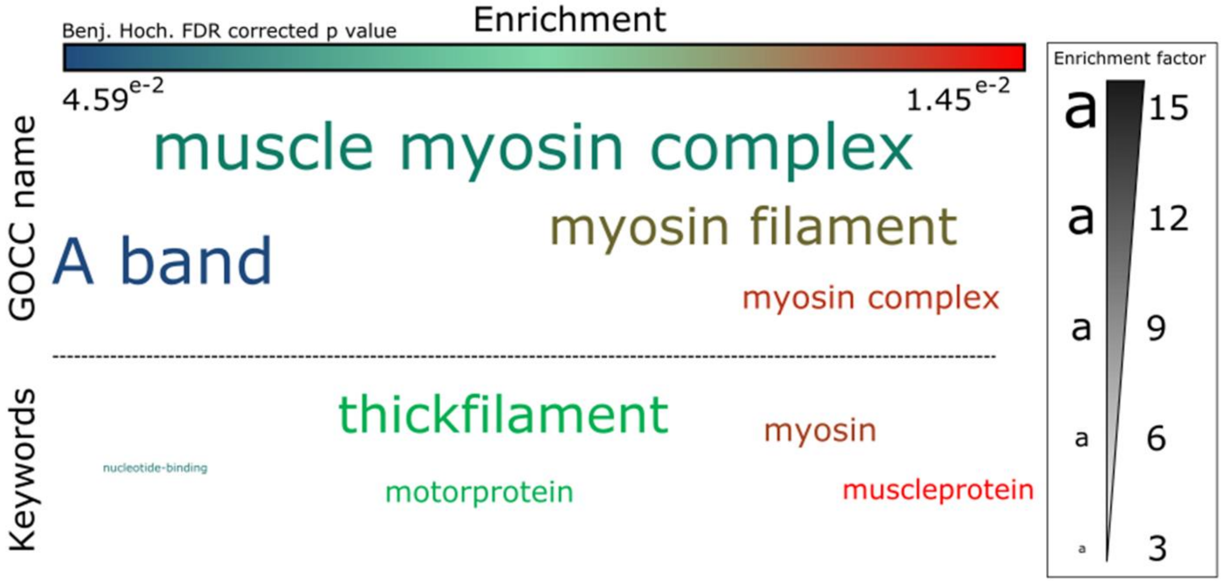

Figure S3 Enrichment analysis of GOCC terms and Uniprot keywords in proteins with greater expression in control medium samples, using Fisher's exact test represented as a word cloud (D). The size of the word represents enrichment of terms, while colour represents FDR corrected p value. All terms with a minimum 0.05 FDR corrected p value were included.

Pathway description	Observed gene count	False discovery rate (FDR)
GOCC		
myosin filament	6	1.67E-10
myofibril	7	6.42E-06
actin cytoskeleton	8	2.41E-05
muscle myosin complex	3	2.66E-05
contractile fiber	6	1.08E-04
stress fiber	4	2.23E-04
myosin complex	4	2.23E-04
sarcomere	5	5.37E-04
filopodium	4	8.68E-04
intracellular non-membrane-bounded organelle	15	3.03E-03
GOBP		
mesenchyme migration	4	3.24E-07
muscle contraction	6	2.41E-04
muscle filament sliding	3	5.11E-04
mesenchyme morphogenesis	4	5.46E-04
tissue morphogenesis	8	4.41E-03
striated muscle contraction	4	4.56E-03
skeletal muscle contraction	3	8.26E-03
GOMF		
microfilament motor activity	4	3.47E-05
purine ribonucleoside binding	14	3.67E-05
purine ribonucleotide binding	14	3.67E-05
purine ribonucleoside triphosphate binding	14	3.67E-05
small molecule binding	15	2.09E-04
anion binding	15	2.31E-04
motor activity	5	2.42E-04
organic cyclic compound binding	20	3.93E-04
ATP binding	11	8.46E-04
heterocyclic compound binding	19	1.34E-03
Pfam		

Actin	4	1.78E-05
Myosin N-terminal SH3-like domain	3	9.33E-05
Myosin tail	3	3.72E-04
Myosin head (motor domain)	3	1.07E-03
Ribosomal protein L6e	2	1.07E-03
Ribosomal protein L6, N-terminal domain	2	1.07E-03

Table S4 Functional enrichments in Medium proteins using String DB, with observed gene count out of a total 35 proteins with an FDR cut-off of 2%.

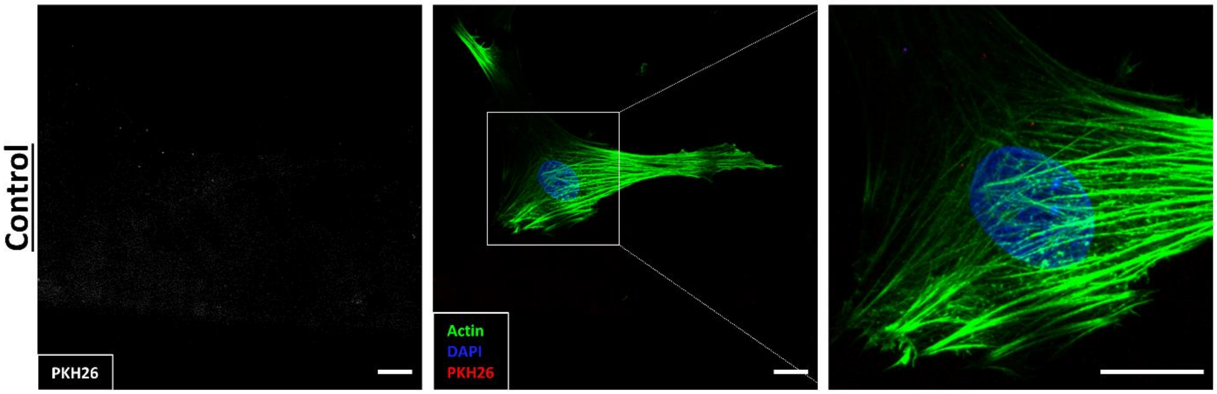

Figure S4 Control samples with no EVs and PKH26 staining demonstrating minimal unspecific fluorescence (Scale = 10μm).
